# Insights into SARS-CoV-2-associated subacute thyroiditis: from infection to vaccine

**DOI:** 10.1186/s12985-023-02103-1

**Published:** 2023-06-21

**Authors:** Mairi Ziaka, Aristomenis Exadaktylos

**Affiliations:** 1 Department of Internal Medicine, Hospital of Thun, Thun, Switzerland; 2 Department of Emergency Medicine, Inselspital, University Hospital, University of Bern, Bern, Switzerland

## Abstract

Since the COVID-19 emergence as a global pandemic in March 2020, more than 5 million SARS-CoV-2-related deaths have been globally documented. As the pandemic progressed, it became clear that, although the infection is mainly characterized as a respiratory disease, it also affects other organs and systems, including the thyroid gland. Indeed, emerging evidence suggests that SARS-CoV-2 can act as a trigger for various thyroid disorders, for example, subacute thyroiditis (SAT), Grave’s disease, and non-thyroidal illness syndrome. The entry of SARS-CoV-2 into the host cells is mainly mediated by the ACE2-receptor, making organs and systems with high expression of this receptor, such as the thyroid gland, highly vulnerable to COVID-19. Accumulating data propose that SAT may be an underestimated manifestation of COVID-19 infection. Importantly, if SAT remains unrecognized, it may trigger or aggravate potential other complications of the disease, for example, respiratory insufficiency and cardiovascular complications, and thus negatively influence prognosis. Moreover, recent case reports, case series, and systematic reviews highlight SAT as a potential side effect of the vaccination against SARS-CoV-2. The present review aims to raise awareness of SARS-CoV-2-associated- and post-vaccination subacute thyroiditis, to discuss recent evidence regarding its pathophysiology, and to present useful information for this special form of SAT related to daily clinical practice.

## Introduction

**Fig. 1:**
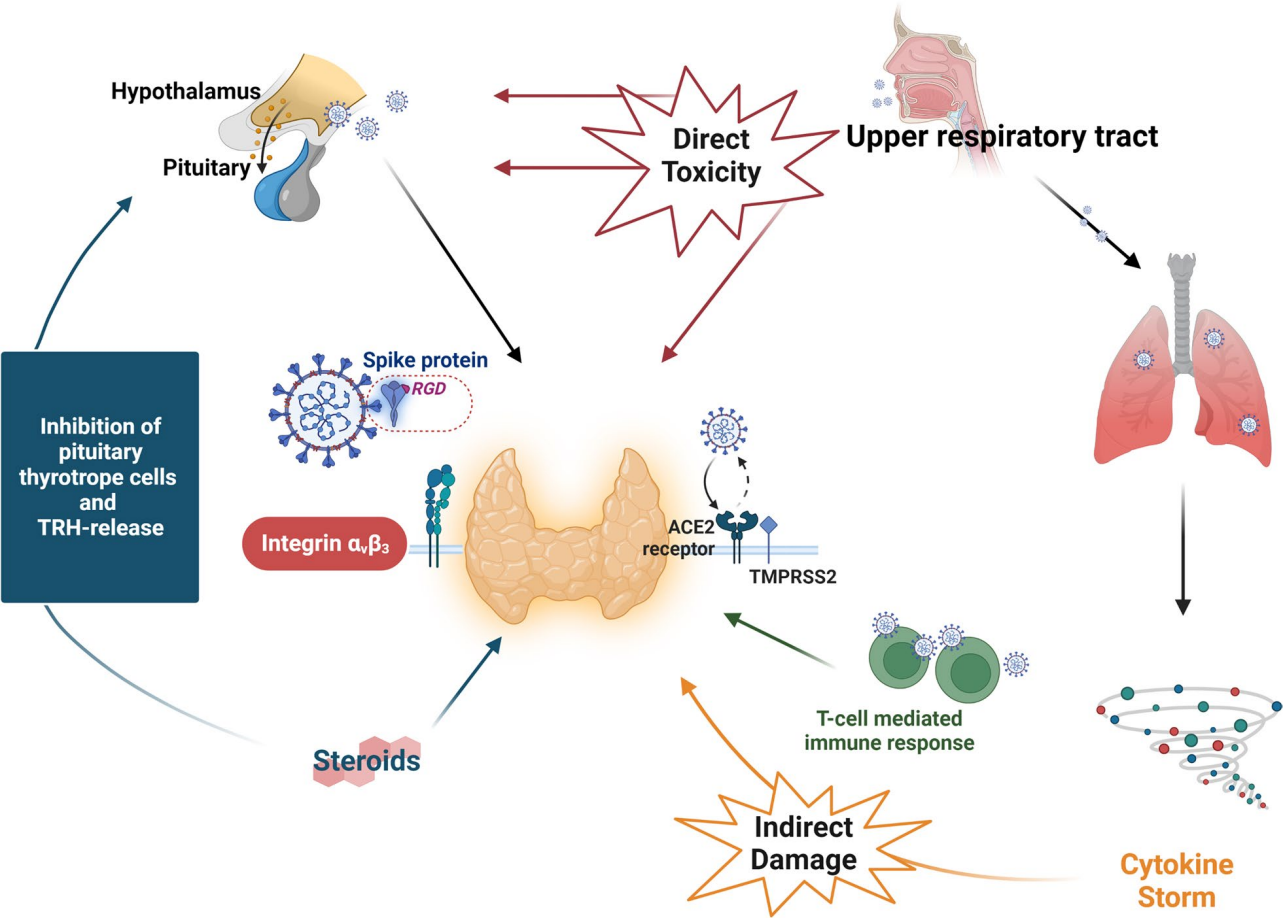
Simplified schematic representation of the main pathophysiological mechanisms of thyroid injury due to COVID-19 infection. SARS-CoV-2 can damage the thyroid gland in a direct and indirect way. Direct toxicity ACE2 and TMPRSS2 on the surface of thyroid follicular cells serve as an entrance gate for the internalization of the virus into the host cells. The same possibly applies to the integrin α_v_β_3_, leading to thyroid gland damage. Additionally, it is suggested that the virus can affect the hypothalamus and the pituitary resulting in HPA-axis dysfunction and, thus, thyroid abnormalities. Indirect toxicity: SARS-CoV-2-induced pulmonary and systemic inflammation and innate immune system activation could potentially damage the thyroid gland in an indirect way. Finally, corticosteroids, which are widely used in the management of COVID-19 patients, inhibit pituitary thyrotrope cells and TRH-release. *ACE2* angiotensin-converting enzyme 2; *COVID-19* Coronavirus disease 2019; *HPA-Axis* hypothalamic–pituitary–adrenal axis; *SARS-CoV-2* severe acute respiratory syndrome coronavirus 2; *TMPRSS2* transmembrane serine protease 2; *TRH* thyrotropin-releasing hormone

The novel coronavirus disease 2019 (COVID-19) caused by severe acute respiratory syndrome virus 2 (SARS-CoV-2) was characterized as a pandemic on March 11, 2020, by the World Health Organization, when 118,000 confirmed cases were diagnosed in 114 different countries [1]. As on April 2021, the number of confirmed cases had exceeded 35 million worldwide, with more than 5 million fatalities making it the most lethal and most rapidly spreading pandemic since the Spanish influenza of 1918 [2, 3]. Despite that the respiratory system represents the main system affected by the virus. This infection can progress to acute respiratory distress syndrome and multiorgan failure; the virus shows a marked tropism for various organs and systems, leading to extrapulmonary manifestations comprising central and peripheral neurological disorders, coagulation disturbances, renal failure, liver abnormalities, cardiac dysfunction including heart failure and arrhythmias, and rhabdomyolysis [4]. Indeed, ACE 2 and transmembrane protease serine 2 (TMPRSS 2), which are variably expressed in human organs and tissues, essentially facilitate the internalization of SARS-CoV-2 into host cells. As we meanwhile know, ACE2 and TMPRSS2 serve as the key molecular complex to enter the host cells and are highly expressed in the small intestine, the kidney, the heart, the brain, and the skin [1]. In addition, the endocrine system demonstrates high ACE2 and TMPRSS2 expression levels, including the reproductive tract, testicle, pancreas, parathyroids, and thyroid gland, making the mentioned glands susceptible to the SARS-CoV-2 infection (Fig. 1) [5].

During the last three years, several studies have been published highlighting the effect of COVID-19 infection on the thyroid gland [5]. Since 2020, some of these studies have been listed in COVID-19-associated SAT, whereas since 2021, SAT has been demonstrated as a complication that can potentially follow SARS-CoV-2 vaccination [6]. Given the increasing incidence of SAT during and post COVID-19 infection and the mass vaccination of the population against SARS-CoV-2, the present review aims to sensitize clinicians to these new clinical entities of SAT, fill the gap regarding its pathophysiology by presenting recent research evidence and quote useful clinical approaches regarding diagnosis and management of SAT in the COVID-19 area.

## SARS-CoV-2-associated subacute thyroiditis

### Pathophysiology

The exact pathophysiology of COVID-19-associated SAT has not been completely deciphered (Fig. 1). However, as mentioned above, direct molecular analysis of surgical samples of thyroid tissues has demonstrated the expression of ACE2 and TMPRSS2 on the thyroid follicular cells making the thyroid gland susceptible to the development of injury during COVID-19 infection either via direct viral toxicity or via an indirect way due to immune-mediated injury or activation of the hypothalamo-pituitary axis by inflammatory cascades [7]. Moreover, another hypothesis regarding the internalization of SARS-CoV-2 into host cells focuses on the role of integrin ανβ3, which recognizes and binds Arg-Gly-Asp (RGD) and Lys-Gly-Asp (KGD), molecular motifs, which are localized in the ACE2 and the spike protein of SARS-CoV-2. Thus, it is hypothesized that integrin ανβ3 may serve as an alternative entrance gate for COVID-19 into host cells [8]. Matters appear to be further complicated by the fact that increased thyroxine concentrations—commonly seen in SAT—induce enhanced expression of integrins, potentially facilitating the entry of the virus into the host cells [1]. Moreover, as it is known, the initial stage of the disease—with its flu-like manifestations and possible transition to pneumonia—is followed by the second stage, characterized by pulmonary and systemic inflammation. Indeed, this phase of the disease commonly coexists with innate immune activation and excessive release of pro-inflammatory cytokines, for example, tumor necrosis factor α, interleukin (IL) 1β, IL-6, and adaptive T-cell-mediated immune responses [7].

Finally, it should be mentioned that genetic predisposition—especially regarding various haplotypes of human leukocyte antigen (HLA)—seems to play a pivotal role in the pathogenesis of SAT [9] via stimulation of aberrant human leukocyte antigen DR isotype expression and the activation of toll-like receptors [10]. The first correlation of SAT with HLA and specifically with *HLA-B35* was carried out by Nyulassy almost 50 years ago [11], a native that was verified in a multitude of publications in the years that followed [9]. In the meantime, it is well documented that 70% of patients with SAT are carriers of *HLA-B35*, while for the remaining 30%, the genetic background is not determined [12]. This observation raises the question if patients who are negative for HLA-B35 have another unknown genetic predisposition. Indeed, further studies have shown that other HLA haplotypes, for example, *HLA-B67, HLA-B15/62*, *HLA-Bw35*, and *HLA-Drw8,* could be additionally implicated in the pathogenesis of SAT [13]. Lastly, a recent, large healthy Polish cohort of Stasiak et al. (2020) highlighted that HLA-B*18:01 and HLA-DRB1*01 represent independent SAT risk alleles and that carriers of HLA-B*35 and HLA-C*04:01 are genetically susceptible to its development [9].

### Clinical, laboratory, and radiologic findings

In the vast majority of cases, SAT is a self-limited inflammatory disease that—although not exclusively—is caused by viral infections [14]. It is presumed to occur either simultaneously with SARS-CoV-2 infection due to direct viral toxicity or to represent a post-viral complication of COVID-19 infection, frequently within 6–8 weeks [6]. Similar to SAT caused by other viruses, COVID-19-associated SAT shows a predilection for middle age female patients [15]. Patients with SARS-CoV-2-induced SAT present classical signs and symptoms caused by thyroid inflammation, including neck tenderness and pain, and symptoms associated with the underlying hyperthyroidism (e.g., fever, palpitations, anxiety, agitation, tremor, heat intolerance, insomnia, weight loss, and excessive perspiration) [15]. However, compared to SAT induced by other infections, some studies have highlighted a stronger intensity of neck pain, a higher incidence of fever, and a higher probability of hypothyroidism at three months of the disease onset [6]. Until March 2022—and two years after the first described case of COVID-19-associated SAT [2, 6]—only 81 cases of SAT related to SARS-CoV-2 infection had been published. This observation raises the suspicion that the described low incidence rate may have been underestimated when the high expression of ACE2 and TMPRSS2 in the thyroid gland is taken into account. Indeed, as mentioned above, neck tenderness and pain constitute the main findings and diagnostic criteria of painful SAT [15]. However, in patients with COVID-19 infection, there is a significant possibility that these signs and symptoms may be either masked by the respiratory symptoms of the disease or modified by therapeutic interventions (e.g., by dexamethasone- and heparin application), leading to atypical forms of SAT and, as such, easily escaping physicians’ attention. Indeed, despite the initial skepticism about corticosteroid administration, almost half of the patients with COVID-19 have been treated with some type of corticosteroid [16]. Moreover, SAT and COVID-19 often present common symptoms (e.g., fever, asthenia, and neck pain), which can lead to misinterpretation of the contemporary presence of thyroiditis [16].

A further diagnostic pitfall in the context of corticosteroid administration is the fact that even low doses of corticosteroid administration can affect TSH levels in humans due to their direct inhibitory effect on pituitary thyrotrope cells and inhibition of the hypothalamic release of TRH. Furthermore, acute glucocorticoid administration results in a decrease in circulating T3 to T4, indicating that these agents hinder the conversion of T4 to T3 [16]. Additionally, in critically ill patients with COVID-19 admitted to high-intensity care units, low levels of TSH and free T3 have been demonstrated in the study of Muller et al. [17]. Moreover, regarding the clinical findings for this specific patients population, the authors described an atypical subtle form of SAT characterized by lymphopenia and not leucocytosis, formulating the hypothesis that the presence of lymphopenia inhibits the pathognomic infiltration of giant cells and, consequently, the appearance of classic symptoms of the disease.

Despite the fact that SAT is mainly a clinical diagnosis, laboratory and ultrasound findings can support its diagnosis further, particularly in atypical manifestations of the disease. Typically, laboratory findings include thyroid function disturbances and elevated inflammatory markers, whereas thyroid ultrasound frequently reveals a voluminous and inhomogenous thyroid gland [6].

### Therapeutic management

Although, to date, no causal treatment for SAT exists, corticosteroids appear to be effective in controlling symptoms even within the first 24 h. In order to avoid the detrimental effects of long-term steroid use, some authors recommend the combined use of steroids/celecoxib. In the case of patients with mild to moderate disease severity, starting with a low dose of prednisolone is encouraged, whereas, for more severe cases, it seems that higher doses cannot be avoided [18].

## Subacute thyroiditis following vaccination against SARS-CoV-2

Similar to infections, vaccination can potentially induce immunologic reactions resulting in autoimmune diseases. The pathophysiological mechanisms are complex and include molecular mimicry, polyclonal activation, epitope spreading, and presentation of cryptic antigenic components [19]. In this context, the development of subacute thyroiditis after vaccination is well established and has been previously described as a consequence of influenza [20], hepatitis B [21], and human papillomavirus vaccinations [22].

Although no curative treatment against SARS-CoV-2 currently exists, various types of vaccines have been developed [3, 23], whose safety and efficacy have been adequately tested in a significant number of clinical studies [19]. Regardless, however, of the provable safety of the vaccines, several types of post-vaccination side effects have been highlighted. The most frequently observed side effect refers to mild reactions, including pain and swelling at the injection site, fever, headaches, chills, muscle/joint aches, and tiredness [24]. However, rare complications, such as thyroid diseases, including SAT, may also occur [19].

**Fig. 2:**
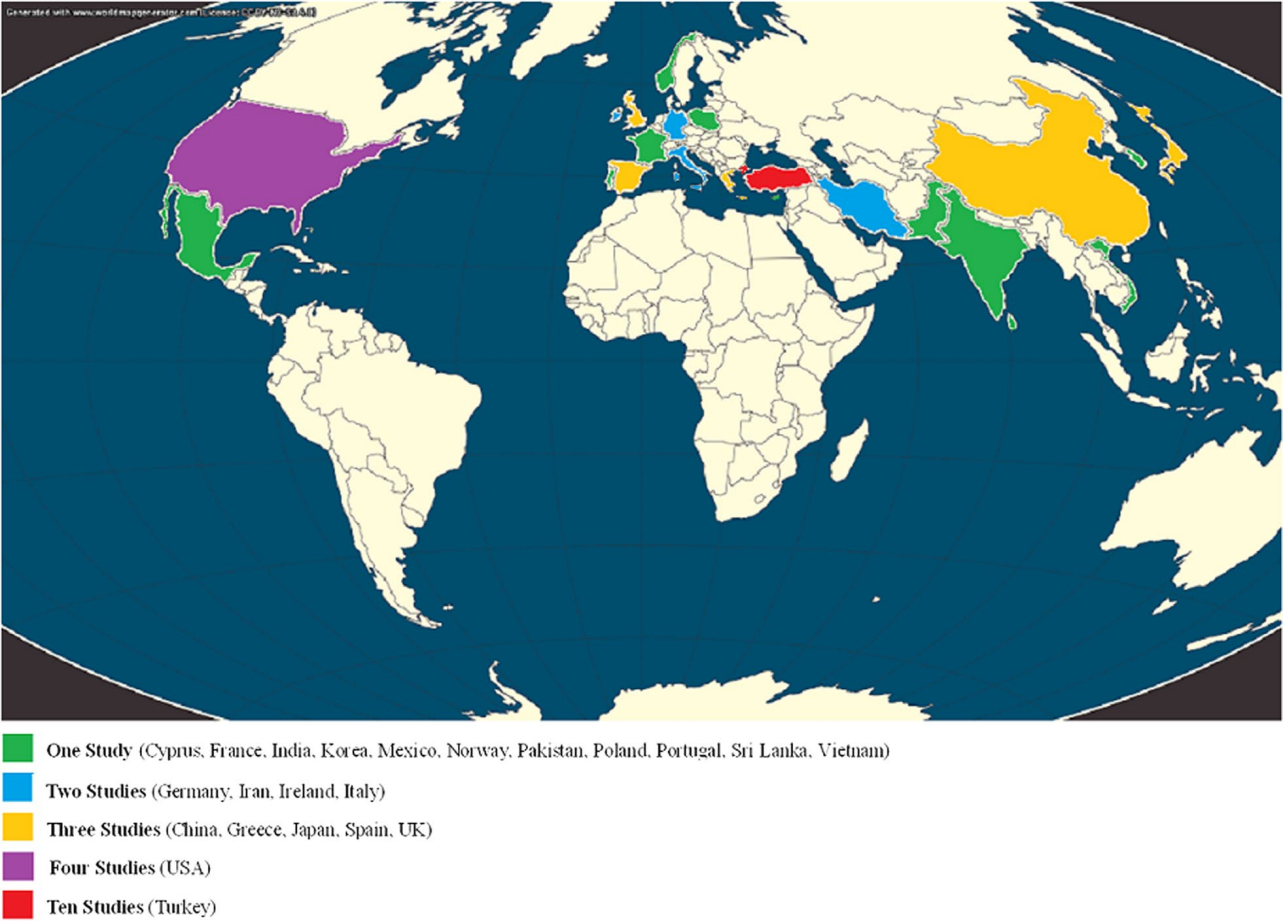
Illustration of the number of studies per country examining post-vaccine SAT

There is mounting evidence from case reports, case series, and retrospective, prospective, and longitudinal studies indicating that SAT is a rare adverse effect that has been observed after vaccination with different types of SARS-CoV-2 vaccines (Table 1, Fig. 2) [25–72]. Even though post-vaccination SAT represents an uncommon complication, given the intensity of vaccinations against COVID-19—until March 2022, a total of almost 11 billion vaccine doses had been globally administered [3]—physicians should be aware of this side effect, which should not influence immunization strategies in any case.

**Table 1:** Studies and reports presenting post-vaccination SAT

Authors	Year of publication	Type of publication	Country of publication	Number of patients
Flores Rebollar	2022	Case report	México	1
Murashita et al.	2022	Case report	Japan	1
Sözen et al.	2021	Case series	Turkey	5
Khan and Brassill	2021	Case report	Ireland	1
Saha et al.	2022	Case report	USA	1
Jhon et al.	2022	Case report	Korea	1
Oyibo	2021	Case report	UK	1
Raashid et al.	2022	Case report	Pakistan	1
González López et al.	2022	Case series	Spain	2
Sahin Tekin et al.	2021	Case report	Turkey	1
İremli et al.	2021	Case series	Turkey	3
Adelmeyer et al.	2022	Case series	Germany	2
Vu et al.	2022	Case report	Vietnam	1
Ie et al.	2023	Case report	Japan	1
Bahçecioğlu et al.	2022	Prospective study	Turkey	6
Chatzi et al.	2022	Case series	Greece	2
Frangos et al.	2022	Case report	Cyprus	1
Casey and Higgins	2022	Case report	Ireland	1
Kishimoto et al.	2022	Case series	Japan	2
Zhao et al.	2022	Case reoprt	China	1
Borges Canha et al.	2022	Case report	Portugal	1
Pujol et al.	2022	Case report	Spain	1
Saygılı and Karakilic	2021	Case report	Turkey	1
Brès et al.	2022	Case series	France	2
Pandya et al.	2022	Case series	USA	3
Siolos et al.	2021	Case report	Greece	1
Pla Peris et al.	2022	Case series	Spain	4
Alkis and Baysal	2022	Case report	Turkey	1
Teti et al.	2022	Case report	Italy	1
Patel et al.	2022	Case report	USA	1
Bornemann et al.	2021	Case series	Germany	2
Ratnayake et al.	2022	Case report	UK	1
Das et al.	2022	Case report	India	1
Jeeyavudeen et al.	2021	Case report	UK	1
Plaza-Enriquez et al.	2021	Case report	USA	1
Vasileiou et al.	2022	Caserepport	Greece	1
Bostan et al.	2022	Retrospective study	Turkey	16
Pi et al.	2022	Case report	China	1
Wijenayake et al.	2022	Case report	Sri Lanka	1
Yorulmaz and Sahin Tekin	2022	Cases series	Turkey	11
Pipitone et al.	2022	Case report	Italy	1
Huang et al.	2022	Case series	China	3
Stasiak et al.	2022	Case series	Poland	2
Topaloğlu et al.	2022	Retrospective study	Turkey	23
Soltanpoor and Norouzi	2021	Case report	Iran	1
Reisi-Vanani et al.	2022	Case report	Iran	1
Sigstad et al.	2021	Case report	Norway	1
Oğuz et al.	2022	Longitudinal study	Turkey	15

### Pathophysiology

As previously mentioned, the pathogenesis of vaccine-induced-SAT is mediated by various pathophysiological mechanisms, for example, molecular mimicry, polyclonal and bystanding activation, epitope spreading, and presentation of cryptic antigen determinants [19]. However, the main pathogenetic mechanisms include molecular mimicry between thyroid components (e.g., thyroid peroxidase peptide sequences) and vaccine antigens—particularly the spike protein—and immune system hyperstimulation [6].

The vast majority of vaccines against COVID-19 encode the S protein of the virus, leading to the production of antibodies, which could potentially play a role in initiating autoimmunity via molecular mimicry mechanisms [35]. Moreover, accumulating data indicate that if the vaccine contains antigenic epitopes, which are structurally similar to autoantigens, then the immune reaction to the vaccination antigen can extend to host cells’ antigens, especially in genetically susceptible individuals [73, 74]. Concerning thyroid tissue antigens, recent research highlights that thyroid peroxidase sequences share structural similarities with SARS-CoV-2-related proteins [35], potently contributing to a cross-reactive immune reaction between the spike protein of the virus and the thyroid antigens [73, 74].

The second major hypothesis regarding the pathophysiology of post-vaccination SAT refers to an autoimmune/autoinflammatory syndrome induced by adjuvants, that is, the ASIA syndrome, according to which the vaccination against SARS-CoV-2 can trigger an autoimmune thyroid response [6]. Indeed, it has been proposed that vaccine adjuvants, used to enhance the immunogenicity of the vaccine, are potential triggers of adverse immune reactions [75]. However, this last hypothesis requires further and in-depth investigation as a number of studies failed to show a causal relationship between vaccine adjuvants and autoimmune pathologic entities [19].

Additionally, it should be mentioned that vaccine-related S protein may directly interact with thyroid cells expressing ACE2 and thus result in thyroid dysfunction [19]. Finally, vaccine-associated enhanced viscosity-status may lead to a pathological release of thyroid hormones from the thyroid, especially in patients with abnormal coagulation status [76].

### Diagnosis and differential diagnosis

Post-vaccination SAT literally refers to a painful thyroid inflammation following vaccination [77]. Consistent with previous studies of SAT of other etiologies [78], recent systematic reviews demonstrate a clear gender preference, with middle-aged women being more affected than men, with a gender ratio of about 2.57:1 [79]. The patient’s age at presentation ranges from 26 to 73 years old [79], while the timeline between vaccination and onset of SAT symptoms lies between a few hours to a few weeks (12 h to 60 days) [77].

Similarly to classic forms of SAT, the most frequently reported signs and symptoms of post-vaccination SAT include anterior neck pain, commonly radiating to the jaw and the ear, neck swelling, headaches, nausea, concentration difficulties, fever, asthenia, fatigue, emotional lability, and signs of thyrotoxicosis (e.g., palpitations, hypertension, weight loss, hyper-defecation, anxiety, and sweating) [79]. It should be noted, however, that in some patients, symptoms caused by post-vaccination SAT may have been underestimated and falsely identified as common vaccination side effects leading to an overlook of the diagnosis of SAT induced by the SARS-CoV-2 vaccine.

Regarding the biochemical characteristics of patients with post-vaccination SAT, thyroid function tests are almost universally consistent with thyrotoxicosis (i.e., suppressed TSH and elevated free T4), with some patients additionally revealing enhanced levels of free T3. Antithyroid antibodies are typically negative, while inflammation markers, such as erythrocyte sedimentation rate (ESR) and C-reactive protein, are typically elevated [51, 79].

Ultrasonographic findings of the disease, when available, include a voluminous, hypoechoic thyroid gland with heterogeneous echo-structure and suppressed vascularization, similar to SAT of other etiologies [77].

A differential diagnosis is of decisive importance for the prognosis of the patients and should include alternative causes of thyrotoxicosis and thyroiditis, such as SARS-CoV-2-associated SAT, as well as causes related to other infectious, for example, Epstein-Barr, cytomegalovirus, influenza, measles, rubella, and mumps [80].

## Conclusions

Nowadays, SARS-CoV-2-associated SAT is considered a rare complication of ongoing COVID-19 infection or a post-viral immune manifestation of the disease. However, accumulating research evidence indicates a high rate of undiagnosed cases of SARS-CoV-2- associated SAT, especially during the acute phase of the disease. Moreover, during the last few years, a variety of studies have indicated SAT as a potential rare side effect of vaccination. However, given the greatest mass vaccination in world history, it is estimated that the number of post-vaccination SAT will undoubtedly increase. High clinical suspicion leading to its early recognition significantly contributes to the improvement of patients’ prognosis. Currently, several issues in reference to the pathophysiology, the clinical presentations, and the specific therapeutic approaches of both SARS-CoV-2-associated- and post-vaccination SAT still remain open. Further research is also needed for the clarification of questions related to the optimization of therapeutic implications and the improvement of prognosis for both pathologic entities.

## Data Availability

Not applicable.
